# Beta-TCP scaffolds with rationally designed macro-micro hierarchical structure improved angio/osteo-genesis capability for bone regeneration

**DOI:** 10.1007/s10856-023-06733-3

**Published:** 2023-07-24

**Authors:** Jianlang Feng, Junjie Liu, Yingqu Wang, Jingjing Diao, Yudi Kuang, Naru Zhao

**Affiliations:** 1 School of Materials Science and Engineering, South China University of Technology, Guangzhou, 510641 PR China; 2 National Engineering Research Center for Tissue Restoration and Reconstruction, South China University of Technology, Guangzhou, 510006 PR China; 3 NMPA Key Laboratory for Research and Evaluation of Innovative Biomaterials for Medical Devices, Guangzhou, 510006 PR China; 4 Medical Devices Research & Testing Center of SCUT, Guangzhou, 510006 PR China; 5 School of Biomedical Sciences and Engineering, South China University of Technology, Guangzhou International Campus, Guangzhou, 511442 PR China; 6 Guangdong Institute of Advanced Biomaterials and Medical Devices, Guangzhou, 510535 PR China

## Abstract

The design of hierarchical porous structure in scaffolds is crucial for bone defect regenerative repair. However, bioceramic materials present a challenge in precisely constructing designed micropores owing to the limitation of forming process. To investigate micropore shape influences bone regeneration in bioceramic scaffolds with macropores, hierarchical porous scaffolds with interconnective macropores (~400 μm) and two types of micropores (spherical and fibrous) were prepared using a combination of direct ink writing (DIW) and template sacrifice methods. Compared to the scaffold with spherical micropores, the scaffold with highly interconnected fibrous micropores significantly improved cell adhesion and upregulated osteogenic and angiogenetic-related gene expression in mBMSCs and HUVECs, respectively. Furthermore, in vivo implantation experiments showed that hierarchical scaffolds with fibrous micropores accelerated the bone repair process significantly. This result can be attributed to the high interconnectivity of fibrous micropores, which promotes the transportation of nutrients and waste during bone regeneration. Our work demonstrates that hierarchical porous scaffold design, especially one with a fibrous micropore structure, is a promising strategy for improving the bone regeneration performance of bioceramic scaffolds.

**Graphical Abstract:**

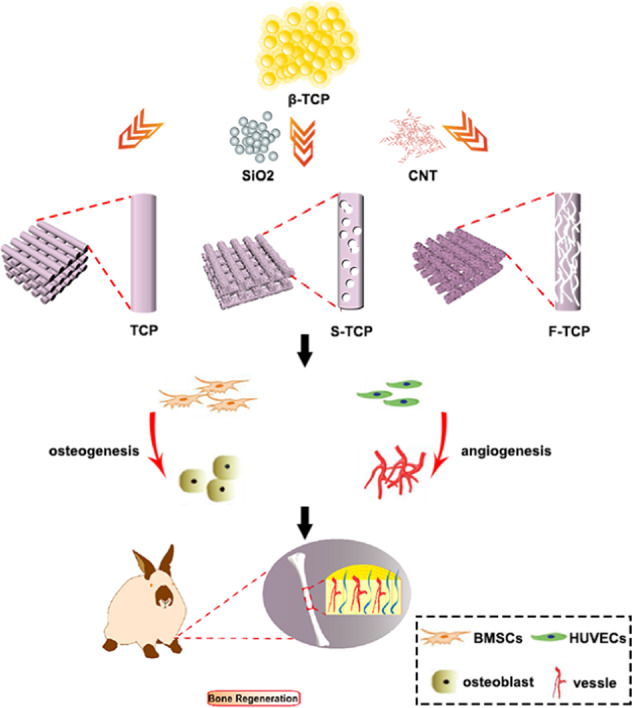

## Introduction

Bone substitute materials are essential for repairing bone defects caused by trauma, disease, and traffic accidents [1]. However, conventional bone substitute materials have limited clinical applications. Autografts, which are considered as the golden standard for bone repairing, are hindered by limited availability and donor-site morbidity. Allografts, on the other hand, have the potential for immunoreactions and disease transmission [2]. Compared to traditional bone substitute, β-tricalcium phosphate (β-Ca_3_(PO_4_)_2_ or β-TCP) has been deemed a promising biomaterial in bone tissue engineering due to its osteoconductivity, degradability, and avalibility [3]. However, conventional β-TCP scaffolds have limited osteogenic capability because of their unoptimized pore structure, which consists of general macropores with hundreds of micrometers [4].

The lack of micropores is believed to be one of the reasons for inefficient bone regeneration, as revealed by anatomical studies of natural bone [5]. Incorporating micropores into scaffolds has been reported to have a significant impact on promoting bone regeneration. The functions of micropores on scaffolds include providing space for cell migration and nutrient transportation [6, 7], and accelerating resorption and local accumulation of ions necessary for mineralization [8]. Hierarchical porous scaffolds with both macropores and micropores have recently been found to play a critical role in regulating cell behavior and bone regeneration [9]. Pei et al. [10] utilized a multiple-step microwave sintering approach to develop a hydroxyapatite scaffold with two types of micropores. Their animal experiments indicated that the presence of these micropores showed obvious osteoinductivity. Similarly, Park et al. [11] employed a molten salt process to prepare porous β-TCP ceramics with a hierarchical pore structure using sodium chloride particles of two different sizes. Their results demonstrated that bioceramics comprising macro- and mesopores could significantly enhance new bone formation and mineralization. Wei et al. [12] prepared a hierarchical micro/macroporous magnesium–calcium phosphate scaffold by incorporating MPC into CPC. In vitro experiments demonstrated that the hierarchical scaffold promote attachment, proliferation, and differentiation of MG63 cells. Additionally, Zhou et al. [13] fabricated scaffolds with 100–800 μm macropores and 1–10 μm micropores through fused deposition modeling techniques and gas foaming, providing innovative opportunities for controlling cell performance within 3D microenvironments. The aforementioned studies have proved that hierarchical porous scaffolds featuring both macropores and micropores could significantly promote bone regeneration. However, the impact of micropore shape on bone regeneration remains unclear. Besides, the precise control of the hierarchical macro and microporous structure simultaneously has not been thoroughly investigated.

**Fig. 1:**
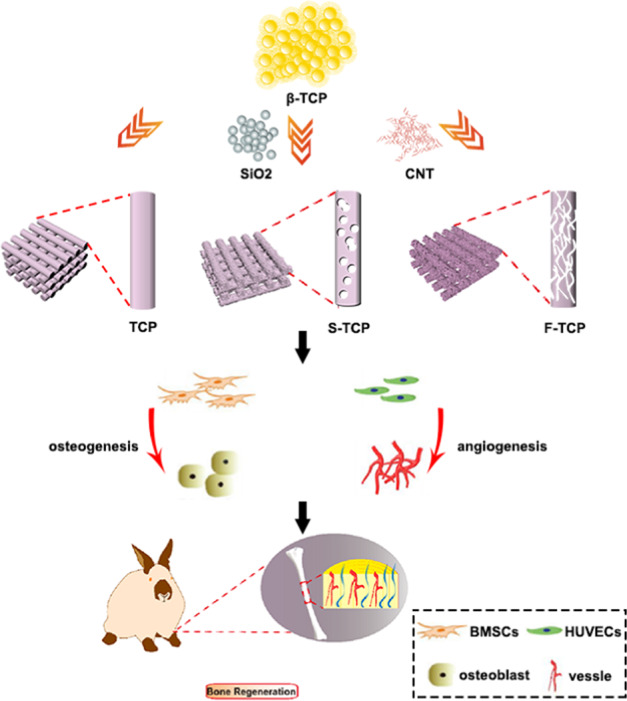
Schematics illustrating the preparation of hierarchical scaffolds and their effect on bone regeneration

In this study, we developed a biomimetic scaffold featuring a hierarchical pore structure. DIW technology was adopted to prepare the scaffold with precisely controlled macropores (~400 μm), followed by a post-treatment step to remove the templates and create micropores within the scaffold. Two different templates, silicon dioxide and carbon nanotube were used to prepare the hierarchical scaffolds with spherical micropores (S-TCP) and fibrous micropores (F-TCP) to investigate the effect of micropore structure on scaffold performance. The resulting differences in pore morphology, porosity, specific surface area, and surface roughness were examined. Furthermore, bone marrow mesenchymal stem cells (mBMSCs) and human umbilical vein endothelial cells (HUVECs) were cocultured with the scaffolds to assess the osteogenic and angiogenic abilities of each scaffold type, as determined by the expression of relevant genes. Finally, we evaluated the bone regeneration performance of the scaffolds in vivo using the New Zealand white rabbit tibia defect model. Our findings demonstrate that scaffolds with hierarchical porous structures, particularly those with fibrous micropores, can significantly accelerate bone regeneration through ion dissolution and protein absorption (Fig. 1).

In conclusion, we have successfully developed a novel approach to constructing both macropores and micropores on β-TCP bioceramic implant simultaneously while adjusting the micropore morphology using two different templates. Besides, microwave sintering process was utilized for bioceramic compaction, thereby eliminating interference from other micropore forms that may arise during conventional sintering. The result demonstrates that hierarchical scaffolds featuring fibrous micropores are highly conductive to the regenerative repair of bone tissue.

## Materials and methods

### Materials

Tetraethyl orthosilicate (TEOS) was purchased from Aladdin (Shanghai, China). Calcium nitrate tetrahydrate Ca(NO_3_)_2_·4H_2_O, ammonium phosphate ((NH_4_)_2_HPO_4_), polyethylene glycol-6000 (PEG, 6000), ammonia monohydrate (NH_3_·H_2_O) and ethanol (CH_3_CH_2_OH) was supplied by Guangzhou Chemical Reagent Factory. All chemical reagents were analytical grade without any further purification.

### Preparation of SiO_2_ sacrificed template

First, 50 mL NH_3_ and 500 mL CH_2_OH were added to 150 mL dH_2_O under magnetic stirring for 1 h. After that, 30 mL of TEOS was added to the mixture and stirred for 6 h at a speed of 45 rpm. Subsequently, another 70 mL of TEOS was added and kept stirred for 12 h. The supernatant was removed after the solution was completely condensed. Finally, the obtained SiO_2_ was washed with deionized water 10–15 times and lyophilized.

### Preparation of β-TCP powder

The β-TCP powder was prepared as described previously [14]. In brief, (NH_4_)_2_HPO_4_ solution (2 M) and Ca(NO_3_)_2_·4H_2_O solution (3 M) were mixed to form β-TCP precipitates at pH 7. The final powder was obtained after being calcined for 3 h at 800 °C.

### Preparation of scaffolds

S-TCP and F-TCP were prepared through DIW combined with templates sacrificial method, while TCP scaffolds were prepared through DIW. The details of preparing scaffolds are described previously [14]. In brief, 21.234 g β-TCP and 1.032 g CNT or 0.8693 g SiO_2_ were mixed by ball-milling to prepare slurry to print F-TCP and S-TCP scaffold. Then the scaffolds were sintered in a microwave furnace. While S-TCP was prepared after being immersed in NaOH (0.2 M) solution.

### Characterization of the scaffolds

To characterize the rheological properties of scaffold paste, a rotational rheometer (MCR302, Anton paar, Austria) was used to measure its viscosity. The measurements were conducted at 25 °C, and the frequency ranged from 0 to 100 Hz. The morphology structure of the scaffolds was observed by field emission scanning electron microscope (SEM, Merlin, ZEISS Inc., Germany). An X-ray powder diffractometer (XRD, Empyrean, Panalytical Inc., Netherlands) with Cu Kα radiation (*λ* = 0.15418 nm) was used to measure the chemical phase of the scaffolds. Atomic force microscope (AFM, NanoMan VS, Bruker Nano Surfaces, Germany) was used to analyze the roughness the scaffolds. The compressive strength of the scaffolds was detected by multi-purpose mechanics testing machine (Instron5960, Instron, USA). The mechanical test of scaffolds (diameter: 10 mm; height: 10 mm) was done on a universal testing machine (ElectroForce3510, America). The scaffold samples were placed between the compression plates. Axial compressive force was placed on the top of the sample. A crosshead speed of 0.5 mm min^−1^ was utilized. The compressive strength was defined as the point at which the compressive stress reached the maximum value.

In order to test the release rate of calcium and phosphorus ions in the scaffolds, the scaffold was immersed in Tris-HCl (pH = 7.4) to test the ion concentration in the solution. Firstly, the scaffolds were washed 3–5 times with distilled H_2_O to remove the ions and other impurities remaining on the surface. Then the mass of each scaffold was weighed and recorded respectively, put 1 wt% Tris-HCl to soak in the scaffold in a constant temperature shaker (37 °C). Take out 50 μL liquid at 0.1, 0.375, 1, 2, 5, 7, 14, 21 day and add equal volume of new Tris-HCl. Finally, determine the concentration of calcium and phosphorus ions in the solution by ICP-OES plasma spectrometer (iCAP PRO ICP-OES, ThermoFisher, USA).

### Cell culture and proliferation assessment

Briefly, mBMSCs (ATCC Inc., USA) and HUVECs (Sciencell Inc., USA) were seeded at the density of 10^5^ per scaffold in 48-well plates for 24 h using complete medium (high glucose Dulbecco’s modified Eagle’s medium (H-DMEM) with 10% fetal bovine serum). Then, the original mediums in the plates were replaced with new mediums. The influences of the scaffolds on the proliferation of mBMSCs and HUVECs were then assessed by using CCK-8 kit (Dojindo Molecular Technologies Inc., Japan) at day 1, 3, and 5. The cell proliferation was detected by CCK-8 kit (Dojindo Molecular Technologies Inc., Japan) after seeding cells onto the scaffold for 1, 3, and 5 d.

### Evaluation of cell adhesion

Cellular adhesion on the surface was observed by confocal laser scanning microscope (CLSM, Leica TCSSP8, Germany). The cytoskeleton was stained by F-actin and the nucleus was stained by 4′,6-diamidino-2-phenylindole (DAPI) after 48 h culture of mBMSCs. Furthermore, the quantitative characterization was measured by detecting the expression of integrin β via western blot. The result of western blot was offered by GuangZhou Lide Biotechnology Co., Ltd.

### Evaluation of protein adsorption

The amounts of protein adsorption were quantified using a BCA protein assay kit (Beyotime, China). 0.2 g scaffold was immersed in 2 mL BSA solution (2 mg/mL) in a constant temperature shaker (37 °C) for 24 h. For the measurement of fibronectin adsorption, 0.2 g scaffold was immersed in 1 mL fibronectin solution (1 μg/mL). Then the scaffold was removed and the amounts of protein was measured. The amount of protein adsorption is equal to the amount of total protein minus the amount of protein in the solution after soaking.

### Evaluation of osteogenesis and angiogenesis in vitro

#### Osteogenic differentiation

qRT-PCR (Chormo4, Bio-rad Inc., USA) was utilized to measure the expressions of typical osteogenesis genes Runx-2, OPN, alkaline phosphatase (ALP) after 7 d and 14 d culture of mBMSCs with scaffold. Glyceraldehyde 3-phosphate dehydrogenase (GAPDH) was used as the endogenous reference gene. Runx-2, OPN, and ALP were detected and the primer sequences are listed in Table S2. Relative expression levels of target genes were determined using the DDCt method. The osteogenic differentiation of mBMSCs was measured using ALP staining. ALP activity was determined by the ratio of ALP enzyme concentration to total protein concentration. Western blotting was performed to quantify ALP and OPN protein levels.

#### Vascularization formation

The expression of eNOS, PDGF-BB, and VEGF was detected as typical angiogenesis genes and the primer sequences are listed in Table S3 The secretion of nitric oxide of HUVECs was detected by NO fluorescence probe DAF-FM DA. Western blotting was performed to quantify CD31 and VEGF protein levels.

### Evaluation of bone regeneration in vivo

#### New Zealand white rabbit tibia defect model and scaffolds implantation

Animal procedures were approved by the Jinan University Laboratory Animal Ethics Committee. 18 male New Zealand white rabbits weighing 2500 g were used to generate tibia defect models. The rabbits were randomly divided into three groups according to the implanted scaffolds: TCP, S-TCP, and F-TCP. Surgery was conducted when the animals were anesthetized. Tibia defects (*Φ* 8 mm × 2.8 mm) were created on the tibia platform. The defect sites were washed with physiological saline solution and implanted with the scaffolds. Suture procedure was conducted in sequence from the innermost (the periosteum) to the outermost (the incision). After that, wound sites were sterilized with iodine solution and wrapped up with sterile gauzes. At weeks 2 and 8, rabbits were euthanized and the samples were obtained. The 3D structure, bone volume/total volume (BV/TV), and trabecular number (Tb.N) of the bone defect areas were obtained via a microcomputed tomography (micro-CT) system (SCANCO MEDICAL μCT-100, SCANCO Medical AG Inc., Switzerland). Histological analyses were performed by cutting the calvarial samples into 4 μm sections followed by H&E and Masson staining.

### Statistical analysis

Quantitative data are expressed as mean ± standard deviation. One-way ANOVA was used for multiple comparisons. A *p* value < 0.05 was considered significant.

## Results

### Characterization of hierarchical scaffolds

The properties of the scaffold paste were analyzed by measuring its rheological behavior, as depicted in Fig. S1. The results indicated that all three pastes displayed non-Newtonian fluid characteristics with a shear-thinning behavior that became more pronounced as the frequency increased, which plays a vitally important role in the DIW process. At low shear rates, the paste exhibited a high viscosity and remain in a static state for shape-preserving. At high shear rates, the paste became more fluid that could be extruded from DIW needle. The incorporation of templates decreased the viscosity of β-TCP paste.

Figure S3 shows the compressive strength of scaffolds. The variation trend of compressive strength of scaffolds was opposite to that of porosity and surface area. The compressive strength of F-TCP scaffolds was 16 ± 4.89 MPa, which was significantly lower than that of TCP (31 ± 8.72 MPa) and S-TCP scaffolds (24 ± 11.58 MPa).

**Fig. 2:**
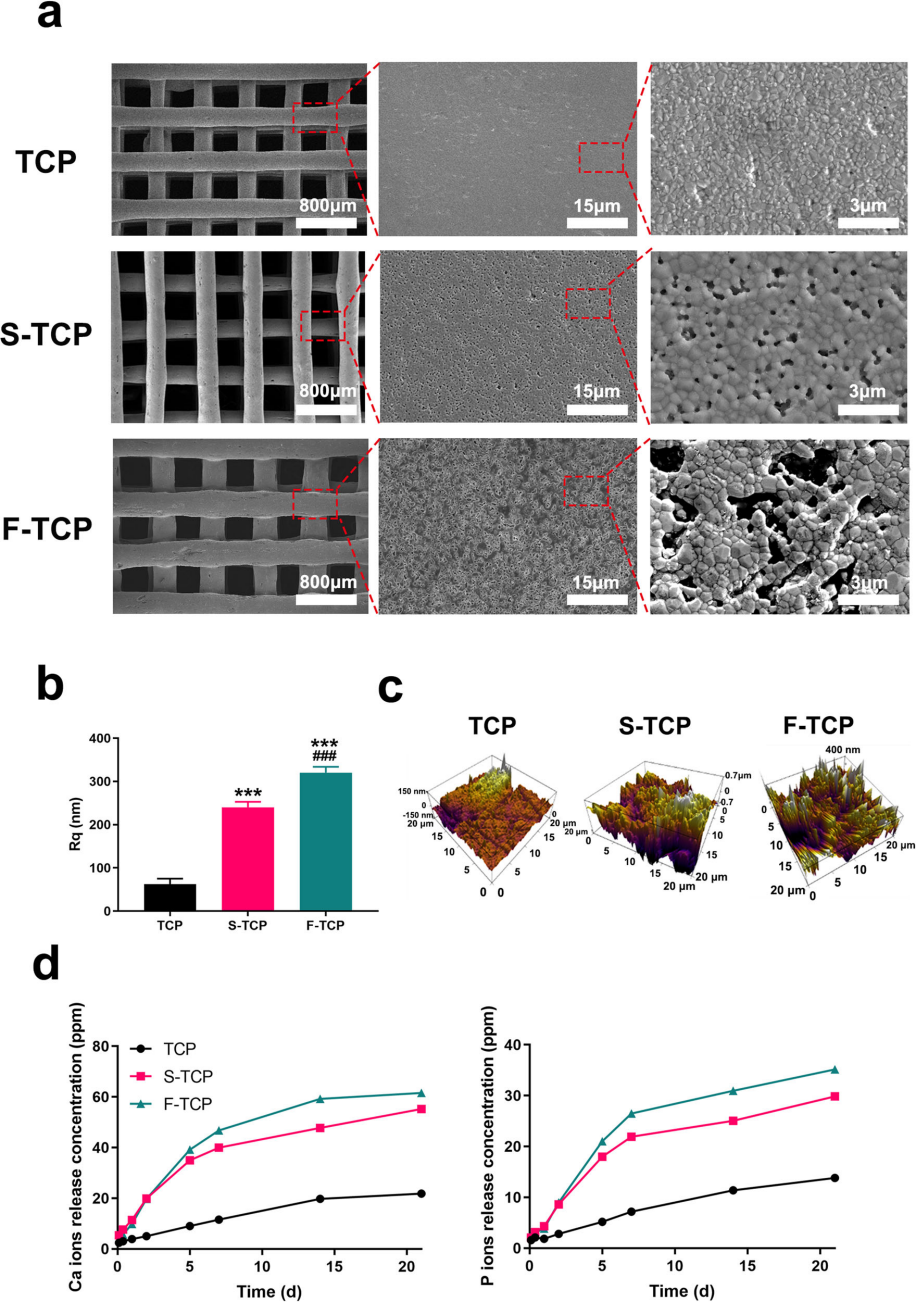
**a** Morphology characterization of the scaffolds analyzed by SEM. **b** The root-mean-squared roughness (Rq) of the scaffolds measured by AFM. **c** AFM images showing the surface topographies of the scaffolds. **d** Cumulative release amount of Ca and P ions of the scaffolds soaked in Tris-HCl. (***p < 0.001 VS TCP scaffold, ###*p* < 0.001 VS S-TCP scaffold)

The macro and microstructure of the scaffolds were characterized via SEM as shown in Fig. 2a. The macropores in each scaffold had a diameter of 400 ± 50 μm, while the micropores, ranging in size from ~500 nm, were uniformly distributed on the struts of S-TCP and F-TCP. The micropores on the S-TCP scaffold were spherical in shape, whereas those on the F-TCP scaffold were fibrous. Conversely, the surface of the TCP scaffold, which solely contained macropore, was compact.

The XRD analysis of the scaffolds was performed to assess their crystalline phase structure, and the results are presented in Fig. S2. The XRD diffraction patterns of TCP, S-TCP, and F-TCP matched the standard JCPDS code of β-TCP.

AFM was used to investigate the surface roughness of the scaffolds. The quantitative analysis revealed that the F-TCP and S-TCP scaffolds were 2–3 times rougher than TCP scaffold, as shown in Fig. 2b, c. The topology of the F-TCP scaffold was significant different from that of S-TCP scaffold. In general, the specific surface area of the three scaffolds followed the diminished sequence below: F-TCP > S-TCP > TCP (Table S1).

To assess the degradation behavior differences among the three scaffolds, the Ca and P ion concentrations in the immersing Tris-HCl solution were measured at different time intervals. As depicted in Fig. 2d, the Ca and P ion release rates of the TCP scaffold were slower than those of the S-TCP and F-TCP scaffolds.

### In vitro cell proliferation, adhesion, and protein adsorption

**Fig. 3:**
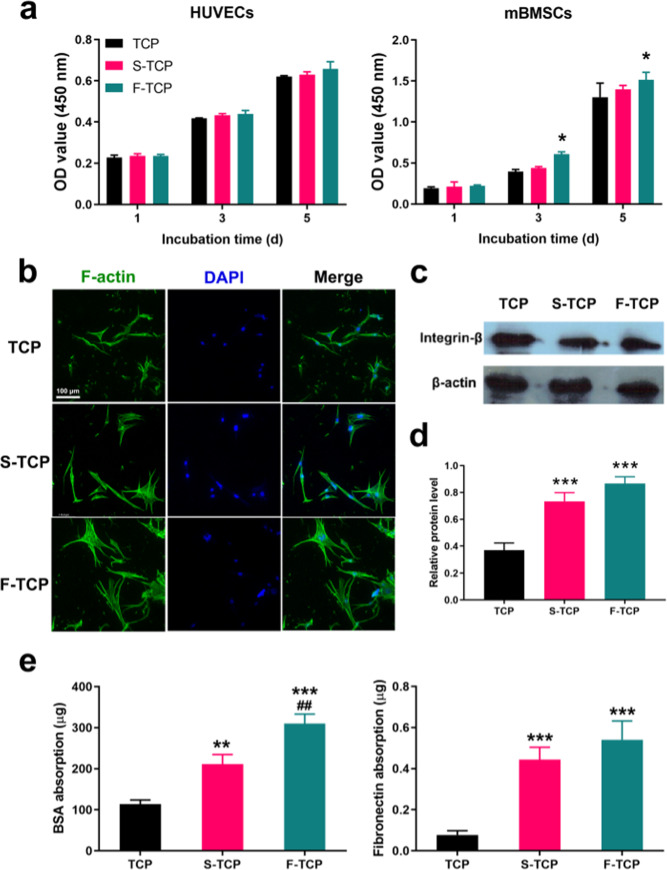
**a** BMSCs, HUVECs proliferation on hierarchical scaffolds. **b** The images of mBMSCs adhering on scaffolds observed by CLSM. **c**, **d** Western blot results of Integrin-β expression in mBMSCs cocultured with hierarchical scaffolds after 48 h. **d** Cumulative release amount of Ca and P ions. **e** BSA and fibronectin absorption of hierarchical scaffolds

To assess the cell viability of mBMSCs and HUVECs, a CCK-8 assay was performed and the results are shown in Fig. 3a. No significant difference was observed in HUVECs co-culture with TCP, S-TCP, and F-TCP at day 1, 3, and 5, indicating good biocompatibility of the scaffolds. mBMSCs proliferation of the F-TCP group is significantly higher than the TCP group when cultured for 3 and 5 days, suggesting that the presence of fibrous micropores significantly promoted the mBMSCs proliferation.

The adhesion of mBMSCs on the scaffolds was tested by CLST. As shown in Fig. 3b, osteoblasts grown on the pure TCP scaffold presented an elongated shape attached to the scaffold surface by filopodia. In comparison, the osteoblast grown on S-TCP showed a longer elongated shape and some of the cells showed a triangle morphology with more filopodia attached to the surface. Interestingly, osteoblasts observed on the F-TCP scaffold showed a polygonal morphology with more outstretched filopodia anchored to the surface.

Moreover, the expression of Integrin-β was quantitatively analyzed by Western blot (Fig. 3c, d), which showed that the expression of Integrin-β was upregulated with the F-TCP and S-TCP scaffolds more than with the TCP scaffold. Those results indicated that F-TCP can effectively promote the adhesion of mBMSCs.

The behavior of protein adsorption was tested by BCA kit. The result (Fig. 3e) demonstrated that compared to the TCP group, the presence of micropores in S-TCP and F-TCP groups significantly improved the adsorption ability of BSA and fibronectin, with the F-TCP group demonstrating the most pronounced effect.

### In vitro osteogenic differentiation

**Fig. 4:**
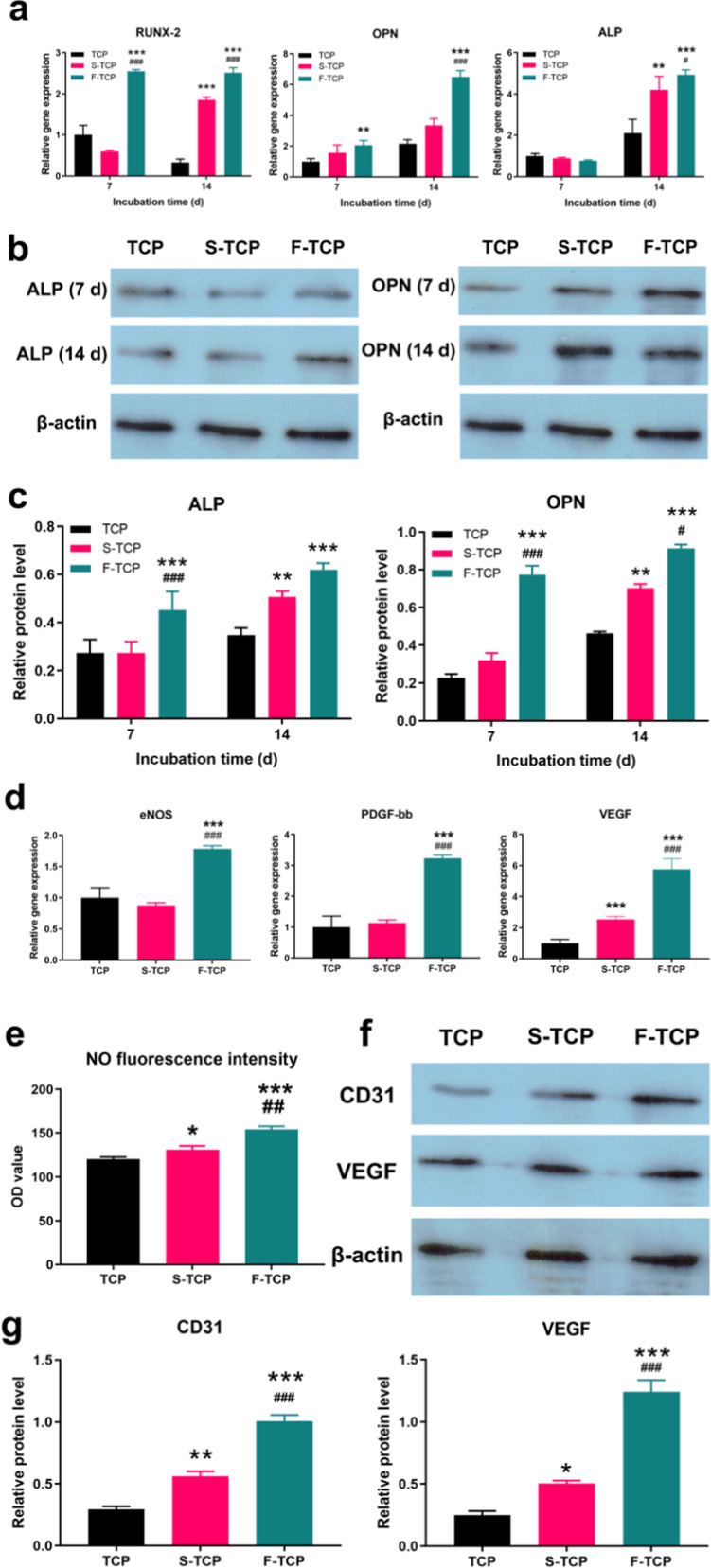
**a** qPCR detection osteogenesis related genes expression of mBMSCs after cocultured with different scaffolds. **b**, **c** Western-bolt analysis of the ALP and OPN protein expression of mBMSCs cocultured with different scaffolds. **d** qPCR detection angiogenesis-related gene expression of HUVECs after cocultured with different scaffolds. **e** NO fluorescence intensity of hierarchical scaffolds. **f**, **g** Western-bolt analysis of the ALP and OPN protein expression of mBMSCs cocultured with different scaffolds

The results of RT-PCR analysis in Fig. 4a indicated that on day 7, the F-TCP group showed significantly higher expression levels of Runx-2 and OPN compared to the other groups. After 14 days of co-culture, the osteoblasts in the F-TCP scaffold exhibited greater up-regulated expression of these predominant genes compared to the other groups. In the S-TCP scaffold, the ALP and Runx-2 mRNA expression levels of osteoblasts were significantly higher than in the TCP scaffold. The protein expression analysis by Western blotting also revealed elevated levels of ALP and OPN in the F-TCP scaffold relative to the other groups (Fig. 4b, c). ALP activity is a crucial indicator for evaluating the osteogenic properties of tissue-engineered scaffolds. As depicted in Fig. S4, the depth of ALP staining in the F-TCP scaffold was significantly higher than in the other groups after 7 and 14 days of co-culture. Notably, there was a significant increase in ALP activity for the S-TCP scaffold after 14 days of co-culture.

### In vitro angiogenic differentiation

The qPCR results indicate a significant improvement in gene expression of eNOs, PDGF-bb, and VEGF in the F-TCP scaffolds after 14 days of culturing compared to the other groups. On day 7, the VEGF gene expression in the S-TCP group was more upregulated than in the TCP groups, as illustrated in Fig. 4d. Western blotting was used to detect CD31 and VEGF protein expression. As depicted in Fig. 4f, g, after 7 days of co-culturing, the F-TCP group exhibited a predominant expression of angiogenic genes compared to the other groups, which is consistent with the direct measurement result of NO fluorescence intensity (Fig. 4e).

### Macro/micro hierarchical porous scaffolds promote bone regeneration in vivo

**Fig. 5:**
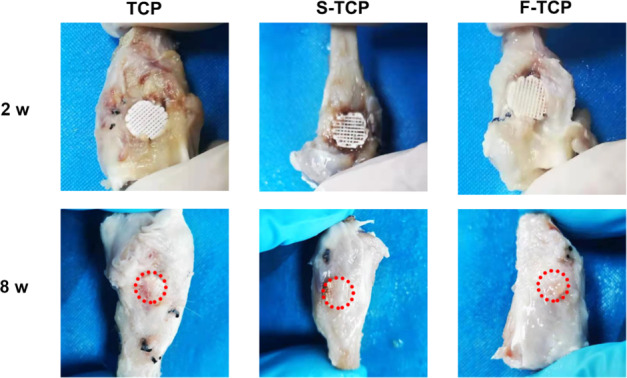
Optical images of retrieved specimens after implanted in tibia defects for 2 and 8 weeks

**Fig. 6:**
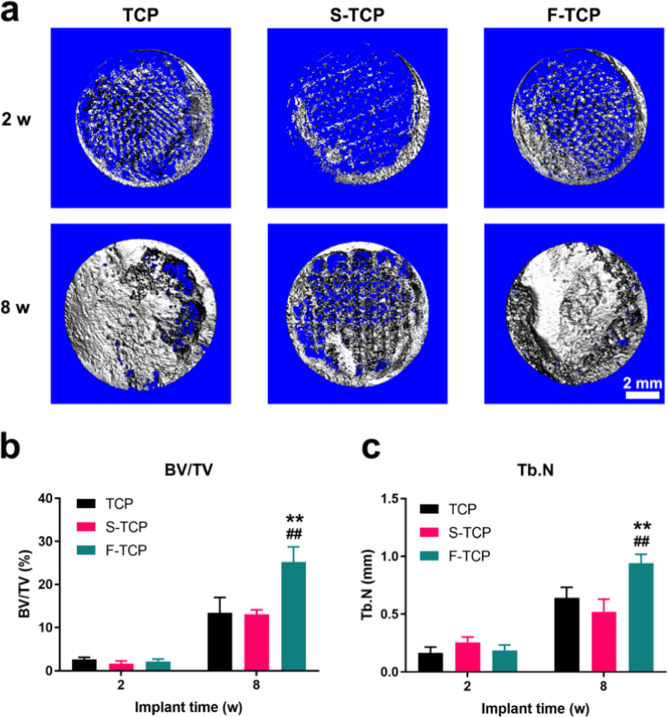
**a** Three-dimensional reconstruction micro-CT. **b** BV/TV quantitative analysis of new bone of the defects. **c** Tb.N quantitative analysis of new bone of the defects

In addition to in vitro studies, we assessed the ability of the hierarchical scaffolds for in situ bone regeneration using the rabbit tibia defect model. All rabbits remained healthy and none succumbed to infection during the feeding process. After 2 weeks of implantation, no abscission of scaffolds was observed, indicating that all scaffolds matched very well with the tibia defect site during the surgical procedures. Additionally, all scaffolds exhibited robust osseointegration, tightly binding to the host bone at 8 weeks, showing great capacity in osseointegration (Fig. 5). 3D micro-CT reconstructed at 2 weeks showed little formation and ingrowth of new bone tissue in all groups. Suboptimal restoration of the tibia defect was observed in the TCP and S-TCP samples at 8 weeks, suggesting inferior bone regeneration compared to F-TCP (Fig. 6a). Statistical analysis indicated that the BV/TV value was significantly higher in the F-TCP group (25.203 ± 3.557%) than in the TCP group (13.464 ± 3.537%) and S-TCP group (13.094 ± 1.067%) at week 8. The F-TCP group also displayed a higher Tb. N value (0.940 ± 0.0785 mm) than the TCP group (0.642 ± 0.089 mm) and S-TCP group (0.520 ± 0.11 mm) (Fig. 6b, c).

**Fig. 7:**
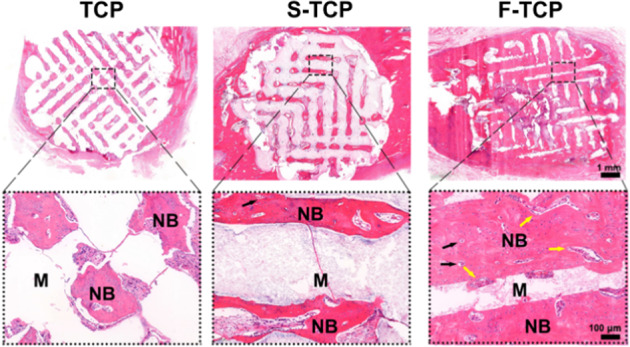
H&E staining of scaffolds implanted in New Zealand white rabbits after 8 weeks (M materials, NB new bone, black arrows indicate Haversian canal, yellow arrows blood vessel)

To comprehensively evaluate new bone tissue growth within the scaffold, we conducted histology studies using H&E and Masson’s trichrome staining. H&E staining (Fig. 7) revealed more blood vessels formed in the F-TCP group after 8 weeks. The edge of the scaffold and surrounding host bone was bound in a large area. The deeper staining of the inner tissues and integrated Haversian canals in the new bone can be observed in F-TCP. Masson’s trichrome staining also showed the presence of numerous mineralized collagen fibers in all three groups after 8 weeks. However, deeper red staining was observed in the F-TCP group, indicating the maturation of newly formed bone tissue (Fig. S5).

## Discussion

At the histological level, bones are categorized into two main structural types - dense cortical bone and porous cancellous or trabecular bone [5]. To promote the process of bone regeneration, biomimicking hierarchical porous structures have been considered a useful method to respond to the microenvironment in vivo. In this study, the sacrificial method combined with DIW technology was utilized to prepare the S-TCP and F-TCP scaffolds. By adjusting the dosage of sacrificed templates, both scaffolds achieved the same level of porosity. The compressive strength of the bioceramic scaffolds decreased with an increase in porosity. Despite this, F-TCP still met the mechanical requirements for a bone defect biomaterial. XRD analysis revealed the crystallographic structure of the scaffolds. The peaks of TCP, S-TCP, and F-TCP were evident at 2*θ* 27.77°, 31.03°, and 34.73°, which were consistent with the XRD pattern of β-TCP (JCPDS 09-0169) (Fig. 3). The XRD patterns of the S-TCP and F-TCP scaffolds confirmed that the chemical composition of the scaffolds was not affected by the introduction of the sacrificed template during fabrication.

The surface roughness of an implant plays a critical role in guiding protein adsorption and cell behavior. Several studies have suggested that a rougher surface is more beneficial for cell attachment and differentiation, which is a key factor in designing successful biomaterial implants for bone regeneration [15, 16]. In our study, the F-TCP scaffold exhibited a significantly rougher surface than the TCP and S-TCP scaffolds. This result was attributed to the introduction of fibrous micropores.

The biodegradation product of β-TCP, which includes Ca and P ions, plays a critical role in the bone regeneration process. An increase in the concentration of Ca ion promotes bone regeneration by upregulating the Wnt/Ca^2+^ signaling pathway [17]. The concentration gradient of Ca^2+^ ions in bone and epidermis triggers a differentiation program after targeting cells to the appropriate tissue locations, which is considered as a chemotactic homing signal [18]. Ca^2+^ ions with high concentration induce chemotaxis of preosteoblast to bone resorption site and promote the regeneration of new bones [19]. Studies have shown that extracellular calcium elicits a chemotactic response from monocytes, osteoblasts, hematopoietic stem cells, and bone marrow progenitor cells by activating the mediation of calcium-sensing receptors [20–24]. Our results indicated that the release of Ca and P ions from F-TCP and S-TCP occurs faster during the degradation period. This phenomenon can be explained by the difference in specific surface area. The hierarchical structure of scaffold improves the contact area between the material and liquid, accelerating the process of ion release and degradation. In summary, the roughness and ion release rate of hierarchical scaffold, especially for F-TCP, were improved. Therefore, we conducted further in vitro and in vivo experiments to evaluate the bone regeneration performance of the hierarchical scaffolds.

Biocompatibility is an essential prerequisite for biomaterials. Therefore, the proliferation of mBMSCs and HUVECs in vitro was first carried out. The CCK-8 assay demonstrated that cells from all three groups survived and proliferated well. These results indicate that the introduction of sacrificed templates did not negatively affect biocompatibility.

The surface morphology of scaffolds is vital in influencing the interaction between cells and the material surface. The adhesion of mBMSCs in the scaffolds were observed using CLSM, where F-actin was used to stain the cytoskeleton, and DAPI was used to stain the nucleus. Our results showed that F-TCP supported the well spreading of mBMSCs on scaffold, which was further supported by the expression of Integrin-β. This can be explained by the larger specific surface area of F-TCP, providing more adhesion sites for cells.

The attachment of proteins to material surfaces requires various transmembrane and extracellular proteins to create attachment sites [25]. Therefore, the surface protein adsorption properties of biomaterial, which are greatly influenced by surface characteristics, play a critical role in regulating cell proliferation, adhesion, and differentiation at the early stages of osteogenesis. Serum protein is the most abundant protein in the plasma and could have an influence on crystal growth in biomineralization by binding with some metal ions and small molecules. Fibronectin is one of the most essential proteins for promoting bone regeneration, as it is involved in actin filament reorganization and cell attachment [26]. Protein adsorption assays demonstrated that the BSA and Fibronectin levels in F-TCP group were significantly higher than those in TCP and S-TCP groups due to the relatively higher specific surface area.

After determining the physicochemical properties and biocompatibility of hierarchical scaffolds, we proceeded to investigate their potential for promoting osteogenic differentiation in mBMSCs through in vitro co-culturing. Specially, we examined the expression of genes related to osteogenesis. Runx-2 is one of the key transcription factors for initiating the process of osteogenic differentiation [27]. Additionally, ALP is known as an early differentiation marker that is upregulated in the initial phases of osteogenesis, while OPN is a late osteogenic marker expressed by mature osteoblasts [28]. Our PCR results indicated that the introduction of micropores, especially for fibrous one, enhanced the gene expression of osteogenic factors, especially in the later stages. Moreover, the production of ALP and OPN proteins was upregulated in F-TCP, and the ALP staining results were consistent with the gene and protein expression. Overall, our findings suggest that the hierarchical scaffolds, particularly F-TCP, have a greater ability to facilitate osteogenic differentiation compared to scaffolds without micropores. The hierarchical structure offers more space for intercellular contacts and paracrine signaling, promoting osteogenic differentiation activity. This favorable osteogenic environment in F-TCP is believed to be attributed to the accumulation of more calcium ions, which accelerates mineral deposition and promotes the expression of osteogenesis-related genes. Therefore, it could be concluded that the osteogenic effect of the F-TCP scaffold is the strongest.

Angiogenesis is a crucial process of osteogenesis, as it not only provides nutrients for cells and tissues but also improves cell activity and migration. Therefore, we analyzed the expression levels of genes involved in angiogenesis. Vascular endothelial growth factor (VEGF) initiates angiogenesis [29], while platelet-derived growth factor-BB (PDGF-BB) recruits pericytes and stabilizes the vasculature [30]. Nitric oxide (NO) is an endogenous product that participates in physiological activities, including regulating vessel relaxation, nerve conduction, platelet aggregation, and immunoregulation [31]. To evaluate the angiogenic effect of hierarchical scaffolds, we selected VEGF, PDGF-BB, and eNOs genes as markers. The result of PCR, western blotting, and NO fluorescence intensity assays suggested that the introduction of irregular micropores played an important role in promoting angiogenesis. The interconnectivity of pores within the scaffolds is a critical factor that affects angiogenesis. The interconnectivity of scaffold has a noticeable effect on the depth and density of vessel invasion [32]. The expression of the angiogenesis-related gene in the F-TCP and S-TCP groups is significantly higher than in the TCP group, which is due to the favorable interconnective environment provided by the micropores. Even though the micropore sizes are not significantly different between the S-TCP and F-TCP groups, the size of the interconnections differs significantly due to structural variations. The study of Xiao et al. [33] revealed that PI3-kinase/Akt/eNOs signal pathway was regulated by the size of the interconnection. The phosphorylation of eNOs and protein kinases was upregulated in the scaffold with a larger interconnection size. When the PI3-kinase/Akt/eNOs signal pathway was blocked by inhibitor LY-294002, the expression of eNOs phosphorylation was decreased, which gives strong evidence that the promotion of angiogenesis is achieved by activating this signal pathway. It is the variation of interconnection size on the S-TCP and F-TCP groups that exhibits distinct differences in the angiogenic capacity. As a result, it can be inferred that F-TCP shows the greatest potential in promoting angiogenesis.

In vivo, the tibia defect model was performed on New Zealand white rabbit. The Micro-CT results showed that the F-TCP group had superior bone regeneration capacity compared to both the TCP and S-TCP groups. Additionally, H&E staining revealed the presence of more blood vessels with evident haemocytes in the F-TCP scaffold, indicating better angiogenesis performance. In 8 weeks, new bone matrix was found surrounding the margin of F-TCP scaffold, suggesting better osseointegration during the process of bone regeneration. The appearance of integrated Haversian canals in the new bone matrix further confirmed that the F-TCP scaffolds group showed the best osteogenesis ability among the three groups. The result of Masson staining reflected that the maturation of new bone tissue in the F-TCP group was significantly higher than that in the other group and was comparable to the intrinsic host bone, which was proved by the almost disappearing of the blue portion of collagen fiber, with red mature bone tissue being the dominant part.

All of these results confirmed the excellent osteogenic and angiogenic abilities of the F-TCP for bone regeneration.

## Conclusion

In this study, we developed hierarchical β-TCP scaffolds with two types of micropores, using CNT and SiO_2_ templates to create spherical and fibrous micropores. Compared to the pure TCP and S-TCP scaffolds, the resulting F-TCP scaffold exhibited high porosity, interconnectivity, and rough surface, which are advantageous for protein adsorption, ion release, and cell attachment. Moreover, the introduction of micropores did not compromise the biocompatibility of the scaffold, while it up-regulated gene expression to enhance osteogenesis and angiogenesis. In vivo studies using New Zealand white rabbit tibia defect model demonstrated that the hierarchical scaffold, especially for F-TCP group, exhibited superior osseointegration and new bone ingrowth. All of the results proved that scaffolds with proper microstructure design are appealing for application in bone regeneration.

## Supplementary information

Supplementary Information
