# Supplementary material for: Beta-TCP scaffolds with rationally designed macro-micro hierarchical structure improved angio/osteo-genesis capability for bone regeneration
Source: J Mater Sci Mater Med. 2023 Jul 24;34(7):36. doi: 10.1007/s10856-023-06733-3 (PMC10366319; doi:10.1007/s10856-023-06733-3)
Supplement: Supplementary file 1 — Supplementary Information [file 10856_2023_6733_MOESM1_ESM.docx]

**Supporting Information:**

Beta-TCP scaffolds with rationally designed macro-micro hierarchical structure improved angio/osteo-genesis capability for bone regeneration

Jianlang Feng, ‡ Junjie Liu, ‡ Yingqu Wang, Jingjing Diao, Yudi Kuang, Naru Zhao


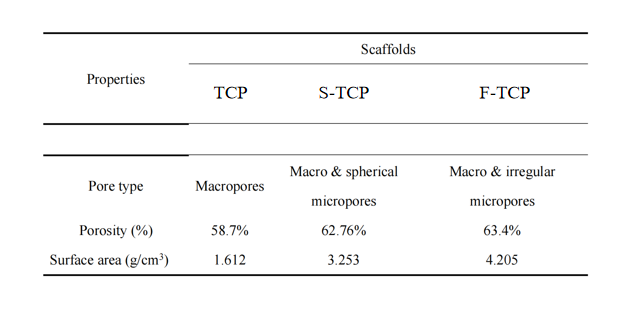


**Table S1.** Textural properties of hierarchical scaffolds.

| Genes | Forward primer (5'–3') | Reverse primer (5'–3') |
| --- | --- | --- |
| *GAPDH* | GATTTGGTCGTATTGGGCG | CTGGAAGATGGTGATGG |
| *RUNX-2* | ATGCTTCATTCGCCTCACAAA | GCACTCACTGACTCGGTTGG |
| *ALP* | GCAAGGGTGAGGAGGGGTA | CCTCTGAAGGCATTTCATAAGCC |
| *OPN* | AGCAAGAAACTCTTCCAAGCAA | GTGAGATTCGTCAGATTCATCCG |

**Table S2.** Osteogenesis-related genes and primers.

| Genes | Forward primer (5'–3') | Reverse primer (5'–3') |
| --- | --- | --- |
| *GAPDH* | GATTTGGTCGTATTGGGCG | CTGGAAGATGGTGATGG |
| *eNOs* | TCAGCCATCACAGTGTTCCC | ATAGCCCGCATAGCGTATCAG |
| *PDGF-BB* | CATCCGCTCCTTTGATGATCTT | GTGCTCGGGTCATGTTCAAGT |
| *VEGF* | GCACATAGAGAGAATGAGCTTCC | CTCCGCTCTGAACAAGGCT |

**Table S****3.** Angiogenesis-related genes and primers.





**Fig S1**. Viscosity of scaffolds paste

**
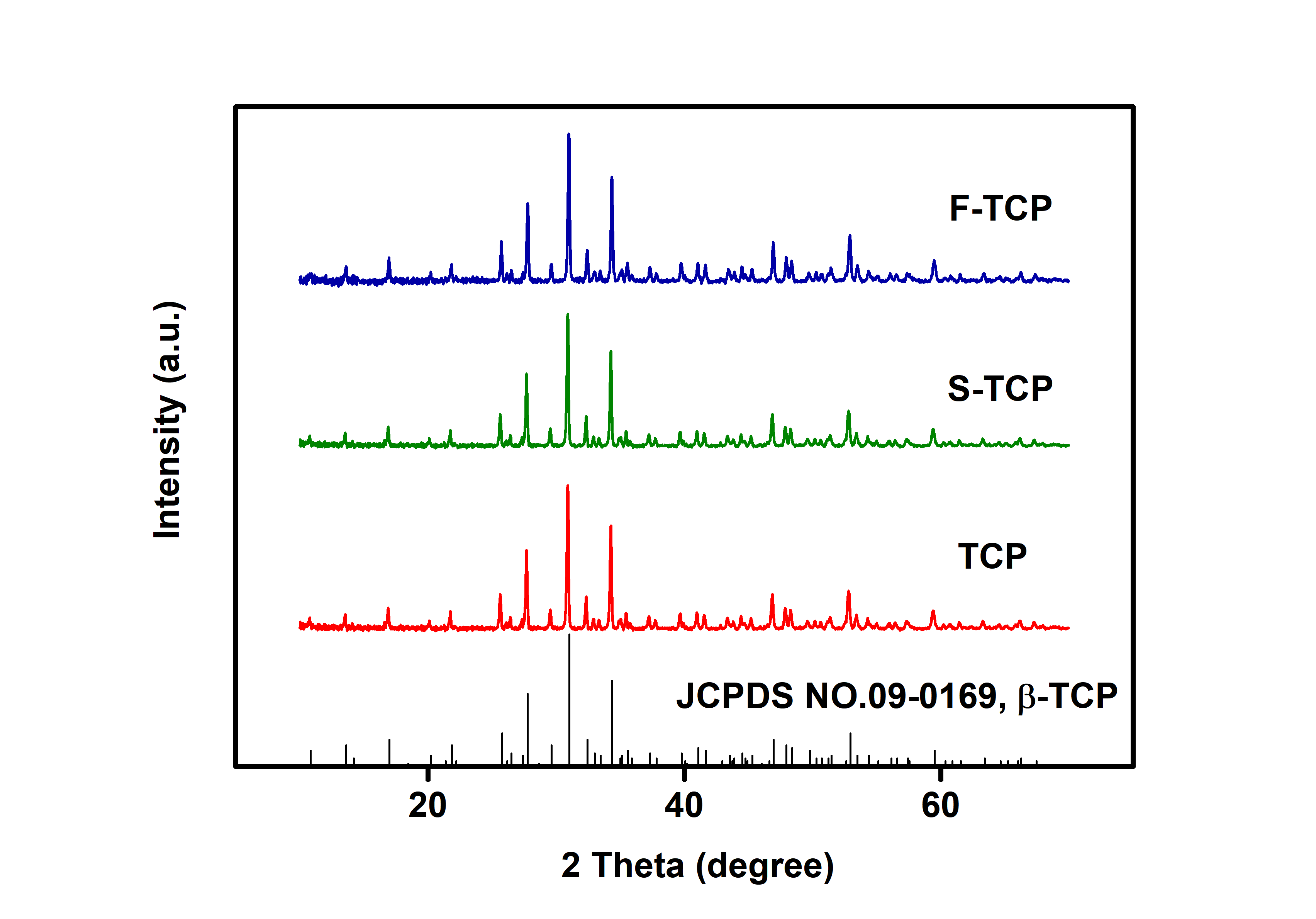
**

**Fig S2**. XRD spectrum of scaffolds


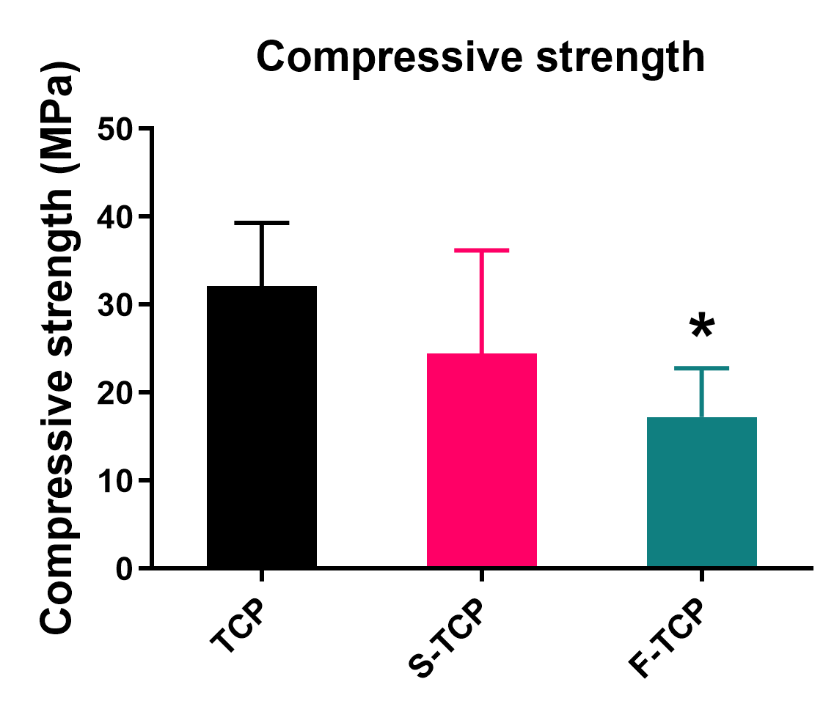


**Fig S3**. The compressive strength of TCP, S-TCP and F-TCP scaffold


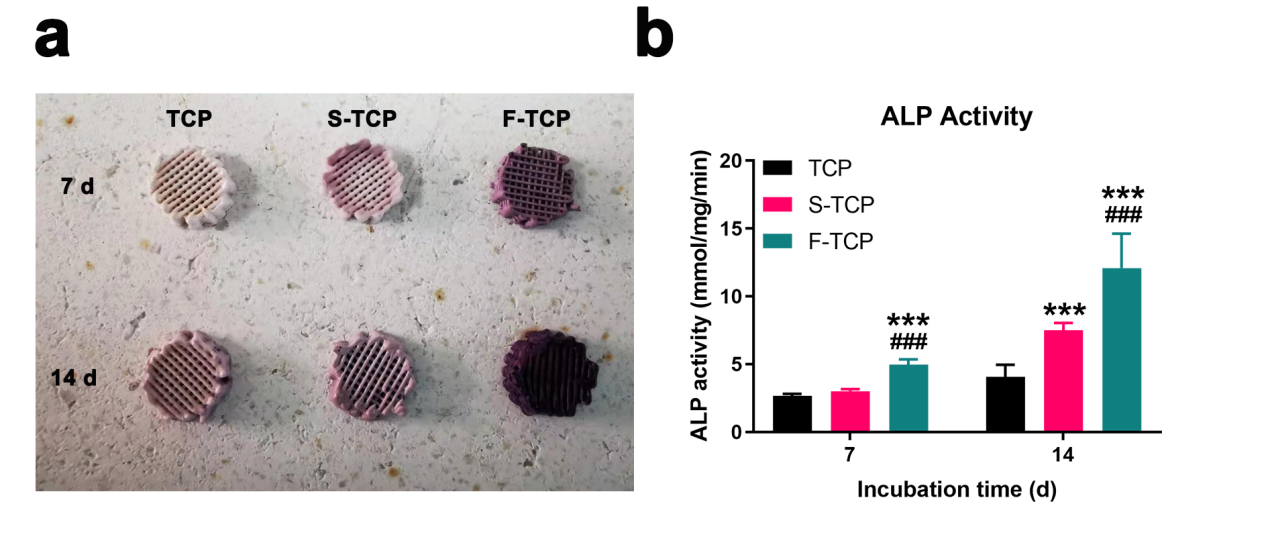


**Fig S4**. The expression of ALP activities of TCP, S-TCP and F-TCP scaffold after 7 and 14 days culturing.


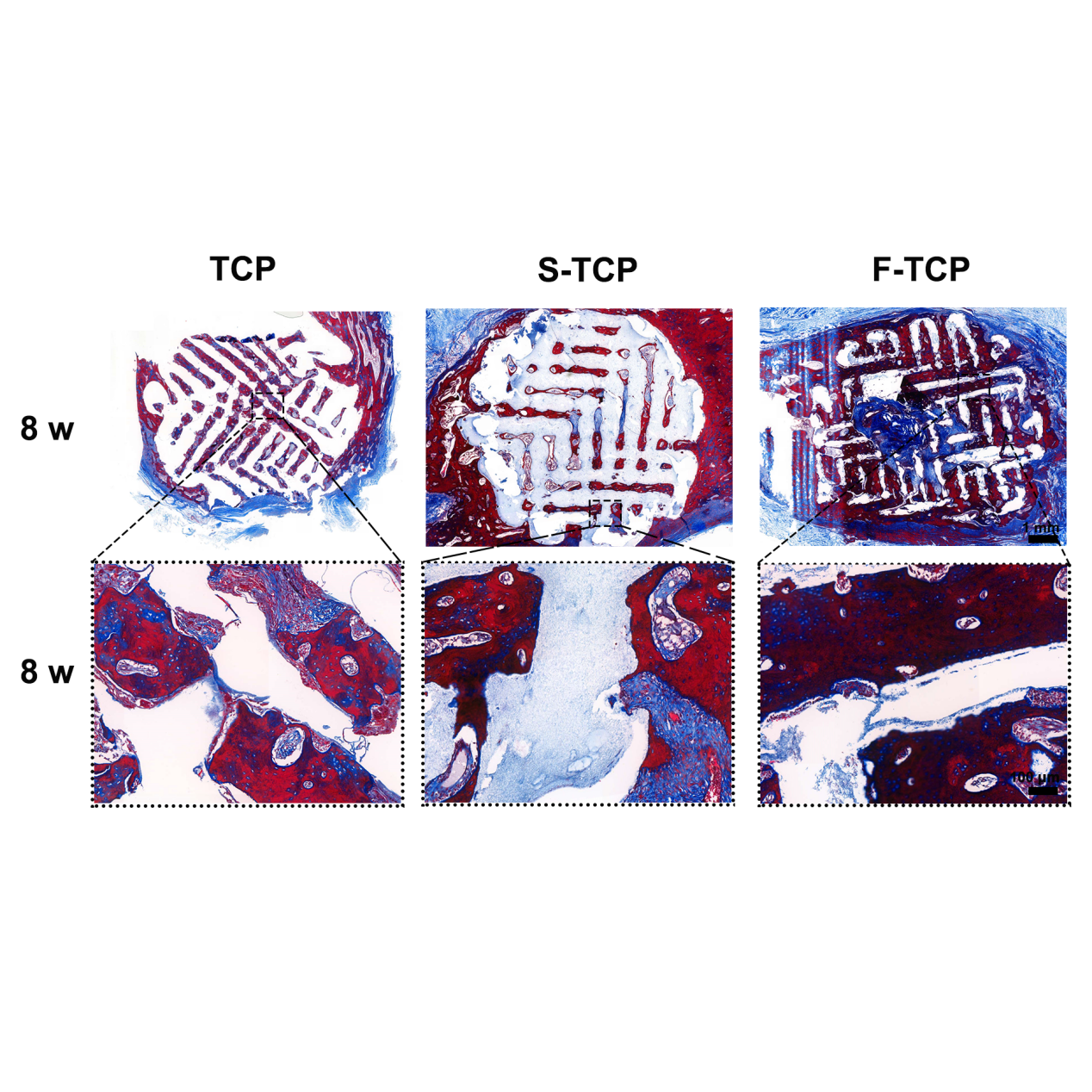


**Fig S5**. Masson staining images of tibial defects after implanted for 8 weeks.
